# Inhibitor-bound complexes of dihydrofolate reductase-thymidylate synthase from *Babesia bovis*

**DOI:** 10.1107/S1744309111029009

**Published:** 2011-08-16

**Authors:** Darren W. Begley, Thomas E. Edwards, Amy C. Raymond, Eric R. Smith, Robert C. Hartley, Jan Abendroth, Banumathi Sankaran, Donald D. Lorimer, Peter J. Myler, Bart L. Staker, Lance J. Stewart

**Affiliations:** a Seattle Structural Genomics Center for Infectious Disease (http://www.ssgcid.org), USA; b Emerald BioStructures Inc., 7869 NE Day Road West, Bainbridge Island, WA 98110, USA; c Berkeley Center for Structural Biology, Berkeley, CA 94720, USA; d Seattle BioMed, 307 Westlake Avenue North, Suite 500, Seattle, WA 98109, USA; e Departments of Global Health, Medical Education and Biomedical Informatics, School of Medicine, University of Washington, Box 357230, Seattle, WA 98195, USA

**Keywords:** *Babesia bovis*, dihydrofolate reductase, thymidylate synthase, pemetrexed, raltitrexed, antifolates, SSGCID

## Abstract

Babesiosis is a tick-borne disease caused by eukaryotic *Babesia* parasites which are morphologically similar to *Plasmodium falciparum*, the causative agent of malaria in humans. Like *Plasmodium*, different species of *Babesia* are tuned to infect different mammalian hosts, including rats, dogs, horses and cattle. Most species of *Plasmodium* and *Babesia* possess an essential bifunctional enzyme for nucleotide synthesis and folate metabolism: dihydrofolate reductase-thymidylate synthase. Although thymidylate synthase is highly conserved across organisms, the bifunctional form of this enzyme is relatively uncommon in nature. The structural characterization of dihydrofolate reductase-thymidylate synthase in *Babesia bovis*, the causative agent of babesiosis in livestock cattle, is reported here. The apo state is compared with structures that contain dUMP, NADP and two different antifolate inhibitors: pemetrexed and raltitrexed. The complexes reveal modes of binding similar to that seen in drug-resistant malaria strains and point to the utility of applying structural studies with proven cancer chemotherapies towards infectious disease research.

## Abstract 2

Structural characterization of the bifunctional enzyme dihydrofolate reductase-thymidylate synthase from *B. bovis* in the apo state and complexed with antifolate inhibitors in both enzymatic active sites is reported.

## 1. Introduction

Babesiosis is an infectious disease affecting humans, cattle and other mammals caused by protozoal *Babesia* piroplasms. Although of less impact than malaria on human health, various species of this api­complexan are thought to be the most common form of blood-borne parasite after trypanosomes (Parola & Raoult, 2001 ▶; Hunfeld *et al.*, 2008 ▶; Homer *et al.*, 2000 ▶). Like *Plasmodium falciparum*, the causative agent of malaria in humans, *Babesia* species generate asexual merozoites through binary fission of red blood cells (RBCs) after infection. Both pathogens also develop mature gametocytes in the host, allowing re-infection and sexual reproduction within their respective transmission vectors: *Anopheles* mosquitoes for *Plasmodium* and *Ixodes* ticks for *Babesia* (Parola & Raoult, 2001 ▶; Florens *et al.*, 2002 ▶). Human babesiosis is somewhat regional, consisting mainly of infections with *B. divergens* in Europe and *B. microti* in the Americas. However, the infection of commercial livestock and other domesticated animals with *B. bigemina* and *B. bovis* has been reported on nearly every continent in both temperate and equatorial climates (Bock *et al.*, 2004 ▶). Thus, *Babesia* represents an emerging threat to commercial livestock and an increasing cause for concern in humans, with recent reports of resistance in *B. microti* to azithromycin–atovaquone drug therapy (Wormser *et al.*, 2010 ▶). For these reasons, babesiosis and other tick-borne illnesses have become a priority with the National Institute for Allergy and Infectious Disease (NIAID), prompting research into the development of vaccines and new chemotherapeutics for treatment. Owing to its genetic and morphological similarity to *Plasmodium* species, drugs which have proven effective in treating malaria may play a critical role in this research (Bock *et al.*, 2004 ▶; Brayton *et al.*, 2007 ▶; Homer *et al.*, 2000 ▶; Hunfeld *et al.*, 2008 ▶). Likewise, mechanisms of resistance which have already appeared in malaria owing to various environmental pressures may provide vital information at the outset of designing novel treatments for *Babesia* infections.

**Figure 1:**
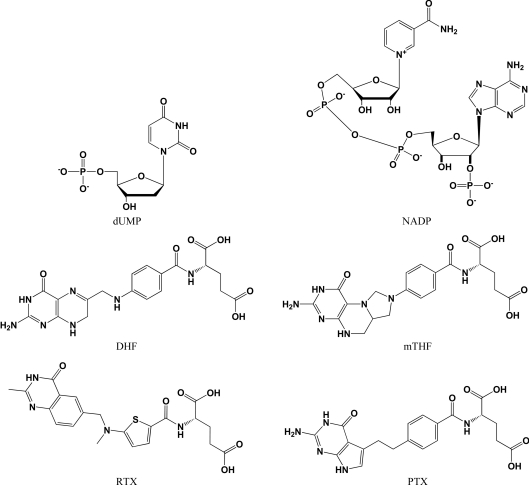
Substrates and cofactors of dihydrofolate reductase (DHFR) [dihydrofolate (DHF) and nicotinamide adenine dinucleotide phosphate (NADP), respectively] and thymidylate synthase (TS) [5′-deoxyuridine monophosphate (dUMP) and *N* ^5^,*N* ^10^-methylene-5,6,7,8-tetrahydrofolate (mTHF), respectively]. Also shown are the two folate analogs and TS inhibitors raltitrexed (RTX) and pemetrexed (PTX).

In this work, we describe the structural features of dihydrofolate reductase-thymidylate synthase from *B. bovis* (BbDHFR-TS), the causative agent of babesiosis in cattle. This bifunctional enzyme is inhibited by pyrimethamine and other antifolates in *P. falciparum* (PfDHFR-TS), to which multiple strains of malaria have become resistant (Peterson *et al.*, 1988 ▶; Yuthavong *et al.*, 2005 ▶; Foote *et al.*, 1990 ▶). The enzyme thymidylate synthase (TS) catalyzes the methyl­ation of deoxyuridine 5′-monophosphate (dUMP) to thymidine 5′-monophosphate (thymidine) employing the cofactor *N* ^5^,*N* ^10^-methylene-5,6,7,8-tetrahydrofolate (mTHF) as both a methylene donor and a reductant (Carreras & Santi, 1995 ▶; Friedkin & Roberts, 1956 ▶). The spent cofactor dihydrofolate (DHF) is then passed through dihydrofolate reductase (DHFR) and serine hydroxy­methyltransferase in order to regenerate mTHF (Carreras & Santi, 1995 ▶). Many species possess the means to salvage and replenish thymidine from DNA-degradation products, the action of thymidine kinase being a primary example (Chen & Prusoff, 1978 ▶). However, inhibition of thymidylate synthase is sufficient to toxify cells which are rapidly dividing (Harrap *et al.*, 1989 ▶; Jackman & Calvert, 1995 ▶). We have obtained the apo structure of BbDHFR-TS as well as complexes of this protein bound to raltitrexed and pemetrexed (Fig. 1 ▶), two antifolates with proven activity as cancer chemotherapeutics (Jackman & Calvert, 1995 ▶; Jackman *et al.*, 1991 ▶; Taylor *et al.*, 1992 ▶). Using this trio of structures, we discuss key residues in BbDHFR-TS which accommodate small molecules and reveal different conformational states of the bound inhibitors in the DHFR subunit. These structures may provide a useful starting point for DHFR-TS drug design and currently comprise a significant structural contribution to the Protein Data Bank (PDB; Berman *et al.*, 2000 ▶, 2003 ▶) for *B. bovis* gene products.

## 2. Methods

### 2.1. Expression and purification

Dihydrofolate reductase-thymidylate synthase (BaboA.01191.a; XP_001609606.1) from the Texas T2Bo isolate of *B. bovis* (Brayton *et al.*, 2007 ▶) spanning the full-length protein from residues 1 to 511 was cloned into a modified pET28 vector. This construct (BbDHFR-TS) was engineered for protein expression in *Gene Composer* and cloned using Polymerase Incomplete Primer Extension (PIPE) cloning into a vector engineered to donate an amino-terminal 6×His-Smt tag with a protease cleavage site to the ORF (Lorimer *et al.*, 2009 ▶; Klock & Lesley, 2009 ▶; Mossessova & Lima, 2000 ▶). The final DHFR-TS protein contains MSHHHHHHSGEVKPEVKPETHINLKVSDGSSEIFFK­IKKTTPLRRLMEAFAKRQGKEMDSLRFLYDGIRIQADQTPE­DLDMEDNDIIEAHREQIGGS– prior to the N-terminal methionine of the native sequence. BbDHFR-TS was expressed in *Escherichia coli* BL21 (DE3) cells in autoinduction medium (Terrific Broth plus Novagen Overnight Express System 1) in a LEX Bioreactor at 293 K for 65 h. Each of several batches of BbDHFR-TS protein was purified in the same manner. The first batch started from 26 g frozen cell paste and was resuspended in 150 ml lysis buffer {200 m*M* sodium chloride, 50 m*M* l-arginine, 25 m*M* tris(hydroxymethyl)amino­methane (Tris), 10 m*M* imidazole, 0.5%(*v*/*v*) glycerol, 0.02%(*w*/*v*) 3-­[(3-cholamidopropyl)dimethylammonio]-1-propanesulfonate (CHAPS) pH 8.0 with 5 µl Benzonase, 100 mg lysozyme and one EDTA-free protease-inhibitor tablet (Roche) added}. The cells were lysed by sonication (70% power for 3 min) and the solution was clarified by centrifugation at an RCF of 4000*g* for 35 min at 277 K.

The clarified lysate was initially purified by nickel-affinity chromatography using the Protein Maker from Emerald BioSystems (Smith *et al.*, 2011 ▶). The column was washed with wash buffer [200 m*M* NaCl, 25 m*M* Tris, 50 m*M* arginine, 10 m*M* imidazole, 1.0 m*M* tris(2-carboxyethyl)phosphine (TCEP), 0.25%(*v*/*v*) glycerol pH 8.0] and eluted with wash buffer containing 500 m*M* imidazole. Fractions containing the protein were pooled, dialyzed into wash buffer and treated with ubiquitin-like protease 1 (Ulp1) at 1 mg ml^−1^ for every 5 mg protein overnight at 277 K. Ulp1 cleaves the protein between the N-terminal methionine of BbDHFR-TS and the C-­terminal serine of the QIGGS tag sequence, leaving no remnant of the tag on the protein. Samples were passed over a 1.0 ml HisTrap nickel column using a syringe pump to bind uncleaved protein, the cleaved 6×His-Smt tag and 6×His-tagged Ulp1, allowing purified BbDHFR-TS to be collected in the flowthrough. The protein was then dialyzed overnight into size-exclusion (SEC) buffer [200 m*M* NaCl, 25 m*M* Tris, 1 m*M* TCEP and 1%(*v*/*v*) glycerol pH 8.0] and concentrated into a 5 ml volume for additional purification by HiPrep Sephacryl S-100 size-exclusion chromatography. Fractions were analyzed by SDS–PAGE and pooled for highest purity. The BbDHFR-TS protein sample was concentrated, flash-frozen in liquid nitrogen and stored at 193 K in 100 µl aliquots for crystallization experiments.

### 2.2. Crystallization

Initial sitting-drop vapor-diffusion crystallization trials were set up at 289 K using the JCSG+, Wizard, PACT and Cryo Full sparse-matrix screens from Emerald BioSystems and the Index HT sparse-matrix screen from Hampton Research. 0.4 µl protein solution was mixed with 0.4 µl reservoir solution and equilibrated against 100 µl reservoir solution using 96-well Compact Jr plates from Emerald BioSystems. The protein solution consisted of 11 mg ml^−1^ BbDHFR-TS in SEC buffer and crystal conditions were optimized using the Microcapillary Protein Crystallization System (MPCS) from Emerald BioSystems (Gerdts *et al.*, 2008 ▶, 2010 ▶; Christensen *et al.*, 2011 ▶). The BbDHFR-TS crystal used to solve the apo structure (PDB entry 3i3r) was obtained from a crystal card running a microfluidic gradient focused at 20%(*w*/*v*) polyethylene glycol (PEG) 8000 and 100 m*M* *N*-­cyclohexyl-2-aminoethanesulfonic acid (CHES) pH 9.5. Additional crystals of BbDHFR-TS were prepared by sitting-drop vapor diffusion using 0.8 µl drops of a 1:1 mixture of crystallization buffer [20%(*w*/*v*) PEG 8000, 100 m*M* CHES pH 9.5] and a fresh batch of protein at 20 mg ml^−1^ in SEC buffer. Unlike the previous batch crystallized *via* MPCS, the new BbDHFR-TS protein sample yielded crystals by sitting-drop vapor diffusion which retained the cofactor NADP throughout purification. The pemetrexed-bound structure (PDB entry 3k2h) was obtained by soaking preformed crystals of BbDHFR-TS prepared by sitting-drop vapor diffusion in fresh drops consisting of 2.0 m*M* dUMP and 2 m*M* pemetrexed in 100% crystallization buffer, with 20 µl buffer in the reservoir. Despite repeated attempts to soak raltitrexed into preformed crystals of BbDHFR-TS, none yielded high-quality crystals with unambiguous electron density for this small molecule. Therefore, cocrystallization trials were conducted by sitting-drop vapor diffusion at 289 K using Wizard, PACT, JCSG+ and Index HT sparse-matrix screens. Drops were prepared by mixing 0.4 µl crystallant with 0.4 µl solution consisting of 2 m*M* dUMP, 2 m*M* NADP, 5 m*M* raltitrexed and 20 mg ml^−1^ protein in SEC buffer, with 100 µl crystallization solution in the reservoir. The crystals used for structure determination (PDB entry 3nrr) were obtained from crystallization buffer consisting of 20 m*M* magnesium chloride, 100 m*M* HEPES and 22%(*w*/*v*) poly(acrylic acid) sodium salt 5100 at pH 7.5.

### 2.3. Data collection and structure determination

All crystals of BbDHFR-TS were looped and cryoprotected in their respective mother liquors spiked with 25%(*v*/*v*) ethylene glycol prior to freezing in liquid nitrogen for X-ray diffraction experiments. The data set for PDB entry 3k2h was collected in-house using a Rigaku SuperBright FR-E+ X-ray generator with Osmic VariMax HF optics and a Saturn 944+ CCD detector. Data sets for PDB entries 3i3r and 3nrr were collected at the ALS synchrotron on beamlines 5.0.2 and 5.0.1, respectively. Diffraction data were reduced with either *HKL*-2000 (Otwinowski & Minor, 1997 ▶) or *XDS*/*XSCALE* (Kabsch, 1988 ▶, 1993 ▶, 2010 ▶). The apo structure was solved by molecular replace­ment using *Phaser* (McCoy *et al.*, 2007 ▶) from the *CCP*4 suite of programs (Winn *et al.*, 2011 ▶) with molecule *A* of DHFR-TS from *Cryptosporidium hominis* (PDB entry 1qzf; O’Neil *et al.*, 2003 ▶) as the search model. The pemetrexed-bound structure was solved by molecular replacement using the apo structure of BbDHFR-TS as the search model; the raltitrexed-bound structure was solved using the pemetrexed complex in an analogous fashion. All crystal structures were initially rebuilt with *ARP*/*wARP* (Langer *et al.*, 2008 ▶) followed by iterative rounds of refinement in *REFMAC*5 (Murshudov *et al.*, 1997 ▶) and manual model building using the *Crystallographic Object-Oriented Toolkit* (*Coot*; Emsley & Cowtan, 2004 ▶). Water molecules were placed in the model using standard σ-cutoff values and distances from the protein as defined by *Coot*. During refinement, every water molecule in every structure was visually assessed and suspicious waters were removed based on inappropriate distances or σ levels using both |*F*_o_| − |*F*_c_| and 2|*F*_o_| − |*F*_c_| density maps. Each structure was evaluated using *MolProbity* (Chen *et al.*, 2010 ▶) and manually checked by internal peer review prior to structure validation and deposition in the Protein Data Bank (Berman *et al.*, 2000 ▶, 2003 ▶). Data-collection details are listed in Table 1 ▶ and refinement statistics for all three structures are contained in Table 2 ▶, with good geometric fitness according to analysis with *MolProbity* (Chen *et al.*, 2010 ▶). Given the resolution limits for the apo structure, *R*_work_ and *R*_free_ still fall within acceptable guidelines given the disordered regions of the linker groups.

## 3. Results and discussion

### 3.1. Overall structure of BbDHFR-TS

**Figure 2:**
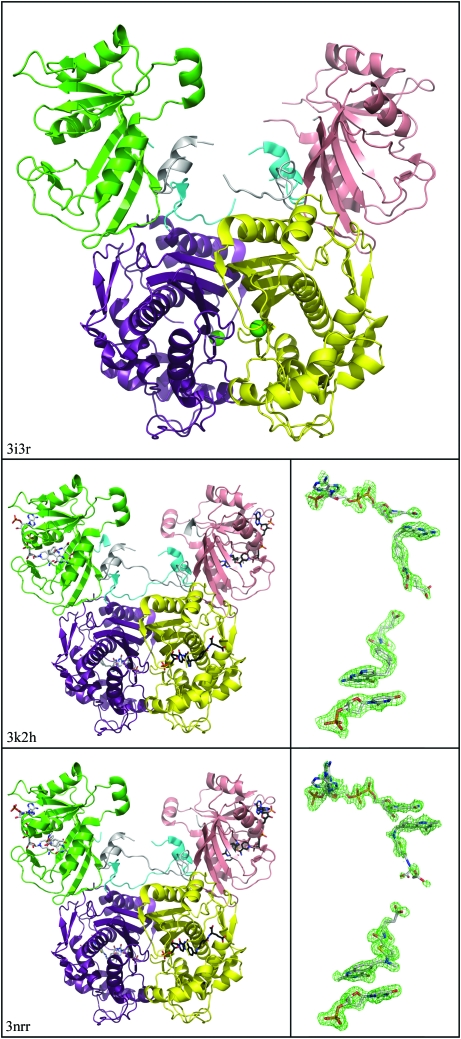
Crystal structures of bifunctional homodimeric dihydrofolate reductase-thymidylate synthase from *B. bovis* (BbDHFR-TS). The DHFR subunit of each protomer (green, pink) is connected to a C-terminal TS subunit (violet, yellow) by a 40-­residue linker (cyan, gray). The protein in the apo state (top; PDB entry 3i3r) has a single chlorine ion in each TS active site (green spheres). Below are structures of BbDHFR-TS bound to dUMP, NADP and pemetrexed (middle; PDB entry 3k2h) and complexed with dUMP, NADP and raltitrexed (bottom; PDB entry 3nrr). Identical ligands are bound to protomer *A* (white, left) and protomer *B* (black, right) in each homodimer complex. Electron density (green mesh) is depicted for protomer *A* ligands at a 2.5σ contour level carved from a 1.6 Å atomic radius of the |*F*_o_| − |*F*_c_| maps using phases calculated from models lacking the ligand. This figure was created using *CCP*4 (Winn *et al.*, 2011 ▶) and *PyMOL* (DeLano, 2002 ▶).

**Figure 3:**
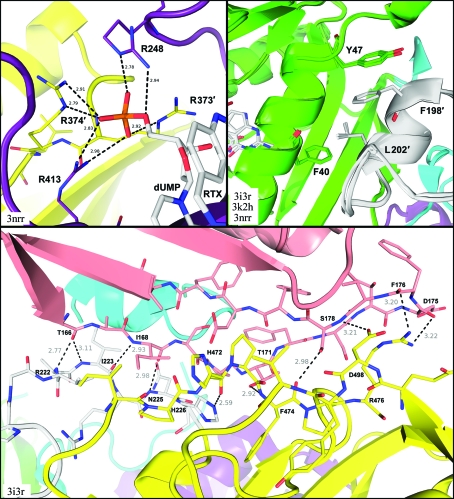
Key subunit interfaces for structures of BbDHFR-TS. Top left, arginine side chains from the opposite TS subunit (yellow) directly bind the terminal dUMP phosphate in the active site of one protomer (violet). Top right, the linker region from one protomer (gray) fits into a hydrophobic groove created by DHFR from the opposite protomer (green). Bottom, binding surface between the DHFR (pink), TS (yellow) and linker (gray) regions of protomer *B* of apo BbDHFR-TS, with labels for hydrogen-bonding residues. All distances are measured in Å. These figures were created using PDB entries 3i3r, 3k2h and 3nrr with *PyMOL* (DeLano, 2002 ▶).

BbDHFR-TS is a homodimer that exhibits the classical dimeric interface seen in other structures of ThyA thymidylate synthase and bifunctional DHFR-TS enzymes (Costi *et al.*, 2005 ▶; Finer-Moore *et al.*, 2003 ▶; O’Neil *et al.*, 2003 ▶; Yuthavong *et al.*, 2005 ▶; Schiffer *et al.*, 1995 ▶). This 511-residue, 58.2 kDa bifunctional enzyme consists of a di­hydrofolate reductase (DHFR) subunit at the N-terminus and a thymidylate synthase (TS) subunit at the C-terminus connected by a linker (Fig. 2 ▶). The first 186 N-terminal residues comprise the DHFR subunit, a nine-stranded β-sheet surrounded by flexible loops and four short α-helices. A 40-residue linker is made of two long flexible chains with a two-turn α-helix in the middle and connects the DHFR and TS domains. The remaining 285 C-terminal amino acids encompass the TS monomer, which consists of a warped four-stranded β-­sheet forming the TS–TS dimeric interface, together with several α-­helices and loops near the active site. BbDHFR-TS is an obligate dimer, with two arginine side chains (Arg373′ and Arg374′) from a flexible loop in one protomer making contact with the terminal phosphate of the substrate dUMP in the opposite protomer (Fig. 3 ▶). Mutational studies have demonstrated these residues are essential for catalytic activity in TS and are 100% conserved across known sequences (Carreras & Santi, 1995 ▶; Michaels *et al.*, 1990 ▶). Two additional arginines from the active-site protomer (Arg248 and Arg413) are also conserved and help to stabilize the deeply buried phosphate of the substrate (Fig. 3 ▶). This highly charged positive cavity in TS often leads to binding of phosphates and other anions present in the crystallization conditions and explains the chlorine ions bound in our apo structure of BbDHFR-TS (PDB entry 3i3r; Fig. 2 ▶).

The DHFR subunits of BbDHFR-TS do not make contact with each other in the quaternary structure and are observed to be approximately 25 Å apart at their closest point. However, the linker from one BbDHFR-TS protomer stretches across to make contact with the DHFR subunit of the opposite protomer (Fig. 2 ▶). This places the enzyme in the ‘long-linker’ family of bifunctional DHFR-TSs together with those of other apicomplexans, such as *P. falciparum* and *C. hominis*, as compared with the short-linker family seen for *Leishmania major* (O’Neil *et al.*, 2003 ▶). The α-helix in the middle of the linker creates a hydrophobic recognition point, fitting into a nonpolar solvent-exposed groove created by Phe40 and Tyr47 on the opposite DHFR subunit (Fig. 3 ▶). Side chains from Leu202′ and Phe198′ on the linker helix fit into this hydrophobic groove, with the latter participating in aromatic stacking with Tyr47 of the opposite protomer. Crystallo­graphically, this interaction remains intact whether BbDHFR-TS is in the apo state or bound to small molecules and may serve to stabilize the holoenzyme. The DHFR subunit also creates a binding surface with the TS subunit and part of the linker of the same protomer. This interface involves a combination of hydrophobic and polar inter­actions between Thr166–Val182 in the terminal edge of the DHFR β-­sheet, Pro219–His226 of the linker and residues Ile470–Arg476 and Glu497–Val500 of TS, all within the same protomer (Fig. 3 ▶). This nonspecific but structural nature of the DHFR-TS interface has been observed previously for bifunctional DHFR-TS enzymes from other species and involves varying contributions from the linker depending on the source organism (Knighton *et al.*, 1994 ▶; O’Neil *et al.*, 2003 ▶; Yuvaniyama *et al.*, 2003 ▶; Yuthavong *et al.*, 2005 ▶).

### 3.2. Comparison of DHFR and TS from *B. bovis* and other organisms

The variability in amino-acid identity for DHFR enzymes from different organisms has afforded opportunities to develop potent drugs with host–pathogen selectivity, such as pyrimethamine and cycloguanil, which are both used to treat malaria (Kompis *et al.*, 2005 ▶; Yuthavong *et al.*, 2005 ▶). However, resistance can arise in response to drug pressure, and mutations in the DHFR subunit of DHFR-TS from *P. falciparum* (PfDHFR-TS) proven to confer resistance to pyrimethamine and cycloguanil include A16V/S, N51I, D54N, C59R, S108T/N and I164L (Foote *et al.*, 1990 ▶; Peterson *et al.*, 1988 ▶; Yuvaniyama *et al.*, 2003 ▶). Four residues (Ile34, Arg42, Thr69 and Leu123) of wild-type DHFR-TS from the Texas T2Bo strain of *B. bovis* (Brayton *et al.*, 2007 ▶) correspond to those seen in a quadruple drug-resistant mutant of PfDHFR-TS. This identity, together with the high structural homology between DHFRs of both organisms, suggest that cycloguanil and pyrimethamine may have low efficacy in targeting DHFR-TS from *B. bovis*.

**Figure 4:**
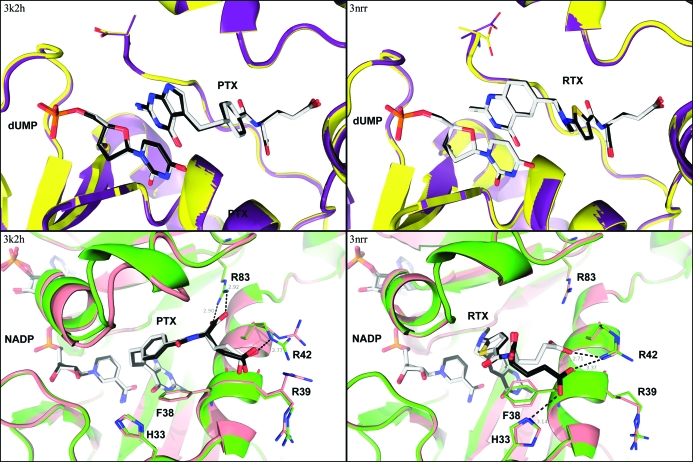
Binding modes of pemetrexed (left) and raltitrexed (right) in the active sites of the TS (top) and DHFR (bottom) subunits of BbDHFRTS. Active sites from protomer *A* (TS, violet; DHFR, green; ligands, white) are overlaid with active sites from protomer *B* (TS, yellow; DHFR, pink; ligands, black) for both complexes. Raltitrexed (RTX) and pemetrexed (PTX) bind the TS active site identically in both protomers of both structures. For the DHFR subunit, the glutamate tail of PTX is consistent across both protomers of 3k2h, creating a salt bridge with Arg83. The glutamate tail of RTX is rotated relative to PTX in the DHFR subunit, resulting in loss of the Arg83 salt bridge, and binds in different conformations to each protomer of 3nrr. This figure was created using *PyMOL* (DeLano, 2002 ▶).

The TS subunit of BbDHFR-TS possesses much higher sequence identity than the DHFR subunit when compared with monomeric DHFR and dimeric TS enzymes from human, yeast and bacteria, as well as other bifunctional DHFR-TS enzymes (Table 3 ▶). The TS domain of BbDHFR-TS possesses a catalytic cysteine (Cys393) which bonds to C6 of dUMP, and a nearby tyrosine (Tyr333) which is purported to abstract the C5 proton from the substrate during catalysis (Carreras & Santi, 1995 ▶). An asparagine which is fully conserved across all eukaryotes is also present in BbDHFR-TS (Asn310); this residue is conserved as a tryptophan in all known prokaryotic sequences. Both Asn310 in eukaryotes and the equivalent tryptophan in prokaryotes appear to perform the same function in TS, which is to bind to the carboxy-terminus and partition off the ternary complex from solvent (Fig. 4 ▶). It has been suggested that a tryptophan in this location does more to orient the folate (and perhaps to exclude larger antifolate inhibitors) than the corresponding asparagine in eukaryotes (Costi *et al.*, 2005 ▶). Wild-type BbDHFR-TS also possesses Ala278 and Ser281, nonconserved residues which usually appear as phenyl­alanine and glycine in other eukaryotes, respectively, whether they be dimeric ThyA or bifunctional DHFR-TS. These residues lie at one end of the cofactor-binding site near the solvent-exposed glutamate tail of mTHF and other folate-like molecules when bound. Alanine and serine at these positions are associated with higher TS reaction rates, as observed for DHFR-TS from *C. hominis* relative to *L. major* and those of other species (Atreya & Anderson, 2004 ▶; Doan *et al.*, 2007 ▶).

### 3.3. Ligand-bound complexes of BbDHFR-TS

We have solved three structures of BbDHFR-TS, one free of ligands and two complexes with fully occupied DHFR and TS active sites. The apo structure of BbDHFR-TS (PDB entry 3i3r) contains no visible substrates or cofactors, only chlorine ions in the arginine-rich phosphate-binding sites for dUMP in the TS subunits (Fig. 2 ▶). The two complex structures of BbDHFR-TS have two ligands bound to each subunit of each protomer in the asymmetric unit. The first complex structure (PDB entry 3k2h) contains the antifolate pemetrexed and dUMP bound in the TS subunit and pemetrexed and NADP bound in the DHFR subunit. The second ligand-bound structure (PDB entry 3nrr) contains raltirexed rather than pemetrexed in both folate sites, as well as dUMP and NADP. The apo state of BbDHFR-TS is structurally similar to the bifunctional enzyme when partially or fully occupied with ligands, but is more disordered. The apo crystal generates higher *B* factors than those of ligand-bound BbDHFR-TS (Table 2 ▶) and has more missing residues than either of the quinternary complexes. The model for the apo structure has three chain breaks per polypeptide chain, two of which are within segments of the linker chain that traverses from one protomer to the other. One of these chain breaks (Lys187–Pro196) is missing from all three BbDHFR-TS structures and appears to be completely surrounded by solvent molecules, making no other contacts with the protein (Figs. 2 ▶ and 3 ▶). The third disordered section of the apo structure comprises a loop which usually contacts the glutamate tail of dihydrofolate (DHF), closing the gate once bound.

Pemetrexed (Taylor *et al.*, 1992 ▶) and raltitrexed (Jackman *et al.*, 1991 ▶) are folate analogs designed as antitumour agents and currently approved for use in some countries for treatment of cancer (Hagner & Joerger, 2010 ▶; Jackman & Calvert, 1995 ▶). Both compounds are nanomolar inhibitors of thymidylate synthase and have been structurally characterized in complex with human TS (Phan *et al.*, 2001 ▶; Sayre *et al.*, 2001 ▶). Like the native cofactor mTHF, all antifolate compounds bind to TS only after the substrate has bound and are sequestered from solvent by a flexible C-terminal tail which closes upon ternary-complex formation (Carreras & Santi, 1995 ▶). Depending on the source organism, raltitrexed has a twofold to 50-fold higher activity against TS *versus* DHFR, while pemetrexed tends to inhibit both enzymes equally well. This unique specificity profile has sparked greater interest in developing potent multitarget antifolates for the treatment of malaria and other infectious diseases (Gangjee *et al.*, 2005 ▶, 2008 ▶; Shih *et al.*, 1997 ▶). When both protomers from each BbDHFR-TS homodimer are compared, the complexed states reveal very similar modes of binding for both drugs to the TS subunit (Fig. 4 ▶). The pteroyl-like groups of pemetrexed and raltitrexed are parallel to the uracil ring of dUMP when bound, mimicking the pteridine ring of the native cofactor mTHF. Although the ϕ angle of the C-terminal Ala511 in one protomer of the raltitrexed complex is rotated by approximately 30° relative to the other, this has no effect on the virtually identical binding modes of raltitrexed in both TS subunits in a single crystal structure. Both drug molecules are also observed in the DHFR subunit but appear to bind this substrate pocket with different geometries (Fig. 4 ▶). The bound conformation of pemetrexed is virtually identical in both DHFR protomers but differs slightly from that of raltitrexed, which itself has two different binding modes for its glutamate tail. The pteroyl-like groups of raltitrexed and pemetrexed again recapitulate the pteridine-ring structure of the native substrate DHF when binding to DHFR. In this case, no structural accommodation is made to differentiate between polar (C2–NH_2_) and nonpolar (C2–CH_3_) scaffold differences, a chemical distinction often associated with the lower DHFR affinity measured for raltitrexed (Gangjee *et al.*, 2008 ▶; Kamen *et al.*, 2000 ▶). However, the *para*-substitution pattern of benzamide in pemetrexed allows the formation of a salt bridge between the α-COOH of its glutamate tail and the Arg83 side chain. This is in contrast to the 2,5-­disubstituted thiophene ring of raltitrexed with no salt bridge to Arg83. The five-membered ring of raltitrexed causes a >90° rotation and a 6.8 Å translation of its α-COOH group relative to pemetrexed (Fig. 4 ▶). Moreover, the glutamate tail of raltitrexed is shifted between protomers of DHFR in the same crystal, with both making hydrogen bonds to Arg42 but only one contacting the N^∊^ atom of His33 (Fig. 4 ▶). These interactions are not seen for pemetrexed, in which the glutamate group is in essentially the same conformation in both DHFR protomers, despite the differences in side-chain rotamers observed for the nearby Arg39 and Arg42. The loss of salt bridge and distinct binding modes of raltitrexed thus provide a structural basis for its lower DHFR affinity relative to pemetrexed, independent of the reduced chemical affinity associated with its C2-methylpteroyl scaffold.

## 4. Conclusions

We have crystallized and structurally characterized DHFR-TS from *B. bovis* in the apo state and in complex with substrates, cofactors and antifolate compounds. The different complexation states of this bifunctional enzyme reveal features similar to those seen in other organisms and confirm its place with the enzymes from other api­complexans in the long-linker family of DHFR-TS. The antifolate drugs pemetrexed and raltitrexed are capable of binding to the DHFR and TS subunits of BbDHFR-TS, indicating a degree of affinity for both drugs to both folate-binding sites. The high sequential and structural homology between DHFR-TS of *B. bovis* and that of *P. falciparum* suggest that effective DHFR-TS inhibitors for malaria may prove to be effective in treating babesiosis. However, mutations which arose in response to drug pressure and have conferred resistance to cycloguanil and pyrimethamine in malarial DHFR-TS already exist as wild-type residues in *B. bovis*. This suggests a reduced chance of such existing drugs working effectively to treat babesiosis by the same mechanism of action. These three structures comprise a significant portion of the *B. bovis* structures currently available in the PDB and provide a structural starting point for rational drug-design efforts aimed at developing novel com­pounds which may lead to new treatments for babesiosis infections.

## Supplementary Material

PDB reference: BaboA.01191.a, 3i3r

PDB reference: 3k2h

PDB reference: 3nrr

## Figures and Tables

**Table 1:** Data-collection statistics for crystal structures of BbDHFR-TS Values in parentheses are for the highest of 20 resolution shells.

PDB code	3i3r	3k2h	3nrr
Space group	*P*1	*P*1	*P*1
Matthews coefficient *V*_M_ (Å^3^ Da^−1^)	2.60	2.56	2.60
Unit-cell parameters			
*a* (Å)	52.54	51.14	51.33
*b* (Å)	83.48	83.20	83.83
*c* (Å)	84.19	83.38	83.92
α (°)	119.0	119.7	119.6
β (°)	98.0	90.9	90.3
γ (°)	100.7	101.7	102.0
Diffraction source	ALS 5.0.2	Rotating anode	ALS 5.0.1
Diffraction protocol	Single wavelength	Single wavelength	Single wavelength
Monochromator	Cryocooled crystal	VariMax HF	Asymmetric curved crystal
Wavelength (Å)	1.00	1.5418	0.97946
Detector	ADSC Quantum 315 CCD	Rigaku Saturn 944+ CCD	ADSC Quantum 315 CCD
Temperature (K)	100	100	100
Resolution range (Å)	72.20–2.35 (2.41–2.35)	50.00–2.20 (2.24–2.20)	50.00–1.80 (1.83–1.80)
Total unique reflections	47995	58064	108796
Completeness (%)	96.8 (96.7)	98.5 (82.6)	97.2 (95.9)
Multiplicity	2.9 (2.9)	4.0 (2.7)	2.0 (2.0)
Mean *I*/σ(*I*)	9.6 (2.2)	15.4 (3.2)	12.3 (2.4)
*R*_merge_†	0.099 (0.551)	0.096 (0.286)	0.060 (0.354)
Phasing method	Molecular replacement	Molecular replacement	Molecular replacement
Starting-model data set	1qzf	3i3r	3k2h

† *R*_merge_ = 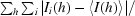 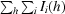.

**Table 2:** Refinement statistics for crystal structures of BbDHFR-TS Values in parentheses are for the highest of 20 resolution shells.

PDB code	3i3r	3k2h	3nrr
Resolution range (Å)	19.69–2.35 (2.41–2.35)	35.20–2.19 (2.25–2.19)	19.94–1.79 (1.83–1.79)
No. of reflections above σ cutoff in final cycle	47995	57443	104245
*R*_cryst_†	0.205	0.192	0.196
No. of reflections for *R*_free_	2440 (181)	2917 (183)	5573 (364)
Final *R*_free_†	0.251 (0.353)	0.239 (0.263)	0.236 (0.270)
Overall average *B* factor (Å^2^)	23.3	16.7	19.8
Average ligand *B* factor (Å^2^)	37.89	25.26	23.31
No. of protein atoms	7607	8072	7965
No. of ligand atoms	2	280	329
No. of solvent atoms	253	727	1025
Total No. of atoms	7862	9079	9319
Residues in favored region (%)	96.5	97.3	98.0
Residues in allowed region (%)	99.7	100	100
Residues in disallowed region (%)	0.3	0.0	0.0
*MolProbity* score [percentile]	1.97 [92nd]	1.69 [96th]	1.36 [97th]

† *R*_cryst_ = 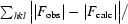 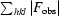. The free *R* factor was calculated using 5% of the reflections, which were omitted from the refinement (Winn *et al.*, 2011 ▶).

**Table 3:** Percentage identity matrix for DHFR (bottom left) and TS (top right) enzymes among species Asterisks (*) denote bifunctional DHFR-TS. Calculated after alignment using *ClustalX* 2.0.10 (Larkin *et al.*, 2007 ▶).

	C. hominis*	P. falciparum*	B. bovis*	T. cruzi*	L. major*	T. gondii*	*H. sapiens*	*S. aureus*	*L. casei*	*E. coli*
Cryptosporidium hominis*		54	57	55	54	55	57	47	50	50
Plasmodium falciparum*	31		63	52	54	61	56	46	46	48
Babesia bovis*	40	33		51	53	53	56	48	49	48
Trypanosoma cruzi*	35	29	27		80	59	63	47	48	50
Leishmania major*	33	25	24	53		59	61	49	49	49
Toxoplasma gondii*	43	28	35	32	32		66	45	45	47
*Homo sapiens*	35	31	33	31	29	37		49	50	54
*Staphylococcus aureus*	31	28	33	25	23	27	28		30	60
*Lactobacillus casei*	25	23	25	22	22	26	24	64		60
*Escherichia coli*	33	28	34	33	30	36	29	41	29	
